# Intradermal Incobotulinum Toxin A for Postbreast Cancer Treatment Asymmetry: A Literature Review and Case Report

**DOI:** 10.1111/jocd.16754

**Published:** 2025-01-03

**Authors:** Alejandra Bugallo, Luis Alberto Parra, Andreina Martinez Amado, Victoria De la Fuente, Evalicia Murúa, Eliana Garcés, Andrea Marcela Parra

**Affiliations:** ^1^ Dermatologist Beauty Care Clinic Buenos Aires Argentina; ^2^ Aesthetics Physician Sociedad Internacional de Rejuvenecimiento Facial no Quirurgico (SIRF) Barranquilla Colombia; ^3^ Aesthetics Physician Clinical Research – Encefalo Bogota Colombia; ^4^ Dermatologist at Levels Clinic in Mexico D.F Mexico Mexico; ^5^ Ophthalmologist – Oculoplastic Surgery Dra Evalicia Murúa Clinic Mexico, D.F Mexico; ^6^ Plastic Surgery Regenera Skin Barranquilla Colombia; ^7^ Ophthalmologist – Oculoplastic Surgery Sociedad Internacional de Rejuvenecimiento Facial no Quirurgico (SIRF) Barranquilla Colombia

**Keywords:** aesthetics, botulinum toxin, breast asymmetry, breast cancer, breast reconstruction, postbreast cancer treatment

## Abstract

**Background:**

Botulinum toxin (BTX) is globally the most common aesthetic procedure. Its usage has expanded beyond facial treatments to therapeutic areas, including managing scars and postsurgical deformities. Breast cancer survivors often face significant deformities and asymmetry during recovery.

**Objectives:**

This study systematically reviewed literature from the past 4 years on botulinum toxin applications in breast cancer survivors and presented a case report of a patient treated with Incobotulinum toxin (IncoBonTA; Xeomin, Merz Pharmaceuticals GmbH, Frankfurt, Germany) for left breast deformity postchemotherapy and radiotherapy.

**Methods:**

Following PRISMA guidelines, a systematic search was conducted on PubMed and Scopus using keywords: “botulinum toxin,” “breast cancer,” and “breast asymmetry,” identifying relevant literature from 2020 to 2024. Five full‐text articles were included. Additionally, a 2024 case report of a patient with significant breast asymmetry postsurgery and radiotherapy was published.

**Results:**

The literature review indicated botulinum toxin's primary uses in breast cancer include pain management, upper limb impairment, postsurgical scars, and capsular contracture. Although some benefits were reported, further research is needed. In the case report, the patient was treated in one session with IncoBonTA at two different dilutions based on contracture severity without complications.

**Conclusion:**

The review showed promising advances in using botulinum toxin for deformities secondary to oncological treatment in breast cancer patients. The therapy was administered to a 53‐year‐old patient, resulting in significant aesthetic improvement, especially at the nipple and areola, suggesting that it was a viable option for these patients.

## 1 Introduction

Breast cancer is currently the most prevalent form of cancer among women worldwide and continues to be the primary cause of female mortality in the United States. Projections for 2040 indicate 684 174 new cases [1, 2]. Over the years, significant advances have also been made in breast cancer research, which has led to more and more treatment options that include surgical intervention, usually mastectomy or breast‐conserving surgery, combined with radiation therapy and chemotherapy [3]. Even though this treatment can effectively control cancer, it results in various complications, like breast asymmetry, deformities, and chronic pain [3, 4].

Despite these challenges, after patients overcome breast cancer treatment, their focus typically goes on achieving aesthetic results by returning to their natural experience. The concept of the “Ideal Female Breast” is a complete study area, especially since breast augmentation is one of the most performed cosmetic procedures in the United States. According to Bekisz et al. [5], moderately sized breasts with a projected contour and fullness in the upper pole are associated with higher attractiveness scores. Specifically, the ideal breast is characterized by a volume distribution ratio of approximately 55:45 between the upper and lower poles, minimal ptosis, and a convex lower pole [5]. But what happens when the breast changes due to mastectomy or radiation therapy? They lead to significant asymmetries with additional loss of breast tissue, making it even more challenging to obtain an aesthetic outcome.

Botulinum toxin type A is gaining popularity as an option to help patients with breast cancer recover from unsatisfactory results after treatment. Currently, various options for botulinum toxin on the aesthetic market all present the exact mechanism of action: inhibition of acetylcholine release at the neuromuscular junction, resulting in temporary muscle paralysis [6].

IncobotulinumtoxinA (Xeomin, Merz Aesthetics North America) is a highly purified form of botulinum toxin type A, with efficacy and safety in dermatological and aesthetic treatments [7]. Additionally, IncoBonTA is being studied for its potential to improve skin quality with injections under the skin, addressing skin quality results [7].

In this case report and literature review, we provide a comprehensive overview of the available information on botulinum toxin A in breast cancer survivors. Additionally, we share our successful experience using Incobotulinum Toxin A injections as a nonsurgical approach to improve breast asymmetry in a patient who underwent breast‐conserving surgery and radiation therapy for breast cancer. Our primary objective was to expand treatment options for these patients by highlighting the potential benefits of IncoBonTA.

### 1.1 Literature Review

This literature review was conducted following the PRISMA (Preferred Reporting Project for Systematic Review and Meta‐analysis) guidelines for conducting a literature review [8]. An online search was performed on PubMed and Scopus databases using the following keywords: “botulinum toxin,” “breast cancer,” and “breast asymmetry,” combining them using the “AND” operator to gather relevant information between 2020 and 2024. Alternatively, the principal focus was clinical trials, review articles, and case reports in English, which present the current uses that involve BoNT‐A inpatients who underwent breast cancer treatment and its outcomes.

In this literature review, we did not evaluate the efficacy of each treatment. We are not comparing management but updating the newest uses for botulinum toxin as an option for breast asymmetry to alter breast cancer.

**FIGURE 1:**
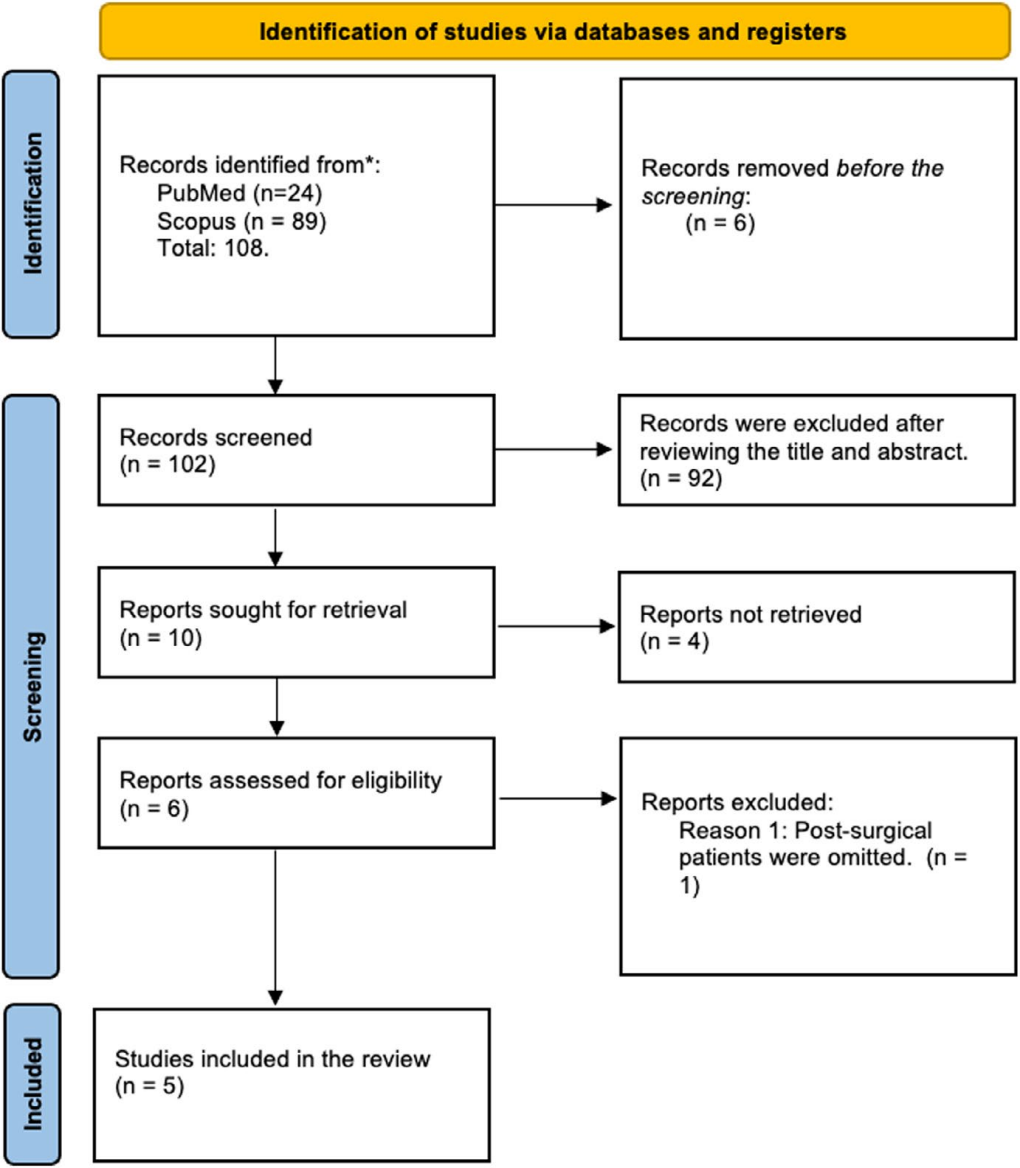
PRISMA flow chart [8].

The initial search yielded 108 papers from all databases. After removing duplicates, non‐English articles, and articles with incomplete data, 102 articles were screened. Following the abstract review, 92 additional articles were excluded, leaving 10 papers for evaluation. Ultimately, five full‐text articles were included in the final analyses (Figure 1) [9, 10, 11, 12, 13].

The selected articles were further analyzed to summarize the current understanding of the use of botulinum toxin in patients who underwent breast cancer treatment. The characteristics of the nine studies are presented in Table 1.

**TABLE 1:** Study characteristics.

Author	Year	Country	Research design	Participants	BoNT‐A indication	Treatment success	Dose(s) used	Plane of application	Follow‐up	Journal
De Groef [10]	2020	Belgium	Double‐blinded, randomized controlled trial	50 breast cancer patients with persistent pain	Upper limb impairments and dysfunctions	No	100 units; Allergan Botox	Intramuscular (pectoralis major)	3 months	Eur J Cancer Care
Aarthi Murugappan [12]	2023	USA	Literature Review	26 papers	Improve postmastectomy pain	Yes	Varied	Peripheral nerve intervention	N/A	Current Oncology Reports (2023)
Aziz D Zikiryakhodzhaev [9]	2021	Russian Federation	Case report	One patient	Capsular contracture after breast reconstruction	Yes	70 UI IncoBonTA	Intramuscular (pectoralis major)	1 week	PRS Global Open
Sen Chen [13]	2020	China	Literature review and meta‐analysis	8 studies	Improve surgical scars	Yes	20–100 Units	Intradermal	Varies	J Craniofac Surg
Waranaree Winayanuwattikun [11]	2023	Bangkok	Randomized controlled study	15 patients	Prevention of postmastectomy scar in transmen	Yes	50 UI IncoBonTA	Intradermal	6 months	Toxins 2023

## 2 Results

Botulinum toxin, a potent neurotoxin produced by the anaerobic bacterium *Clostridium botulinum* , there are seven serotypes of BoNT (indicated with the letters A–G) that distinct, differing in antigenicity and biochemical activity have garnered significant attention in the medical field for their diverse therapeutic applications, including its use [14]. New applications are more popular these days, so this literature review will focus on the emerging role of botulinum toxin in managing aesthetic outcomes in breast cancer survivors [15].

### 2.1 Upper Limb Impairment

Upper limb impairment is a common sequela of breast cancer treatment, and BoNT injections have been used to alleviate spasticity and pain [15]. De Groef et al. [10] reported on the use of botulinum toxin injections into the pectoralis muscle (100 IU) and followed patients for 6 months. Clinical evidence suggests botulinum toxin's efficacy in treating upper limb impairment in breast cancer survivors without negatively affecting upper limb function, range of motion, or pain [10]. This highlights the importance of considering variables such as injection site, dosage, and frequency in future studies and how treatment outcomes should be measured.

### 2.2 Postsurgical Scars

Winayanuwattikun et al. [11] and Chen et al. [13] investigated botulinum toxin (BoNT) for improving postsurgical scars. They found that intradermal BoNT injections (10–100 IU) within 1 cm of the surgical incision improved muscle spasticity and reduced hypertrophic scarring. This simple, well‐tolerated procedure, which requires no anesthesia or hospitalization, offers an attractive alternative to surgical revision. Winayanuwattikun et al. [11] also demonstrated the use of botulinum toxin in transgender patients following mastectomy for gender reaffirmation, resulting in a more masculine chest contour and improved scar development. Poor cosmetic outcomes following mastectomy can significantly negatively impact the mental health of breast cancer survivors, particularly those undergoing total mastectomy [16]. Addressing these aesthetic concerns is, therefore, vital.

### 2.3 Capsule Contracture

Zikiryakhodzhaev et al. [9] reported that using IncoBonTA to address capsule contractures following reconstructive breast surgery could be effective. Applying subdermal injections following a circular pattern around the capsule contracture, using a total of 70 UI, their results showed that administering botulinum toxin injections significantly improved asymmetry, reducing or eliminating the signs and symptoms of capsule contracture.

### 2.4 Pain Management

Lastly, BoNT‐A injections have been recognized as a viable option for addressing postmastectomy pain syndrome, particularly in cases of muscle spasticity and neuropathic pain, providing a minimally invasive intervention with few side effects, Muruguappan et al. [12] presented a well‐written literature review about interventional treatment options for postmastectomy pain management; BonT‐A shows effectiveness in treating incisional pain with neuropathic symptoms. Botulinum toxin can modify the pain process and enhance nociceptive and neuropathic pain by administering multiple injections around the surgical incision area for optimal pain relief.

BoNT‐A may serve as a beneficial supplementary intervention for enhancing the quality of life of patients with breast cancer by addressing both physical and aesthetic concerns following surgical procedures.

## 3 Case Report

This paper presents the case of a 53‐year‐old patient who was diagnosed with breast cancer in 2022; during that year, she underwent an upper right quadrantectomy of her left breast, followed by radiation therapy. After surgery, the patient presented with skin retraction over the operative site, leading to upper breast asymmetry.

**FIGURE 2:**
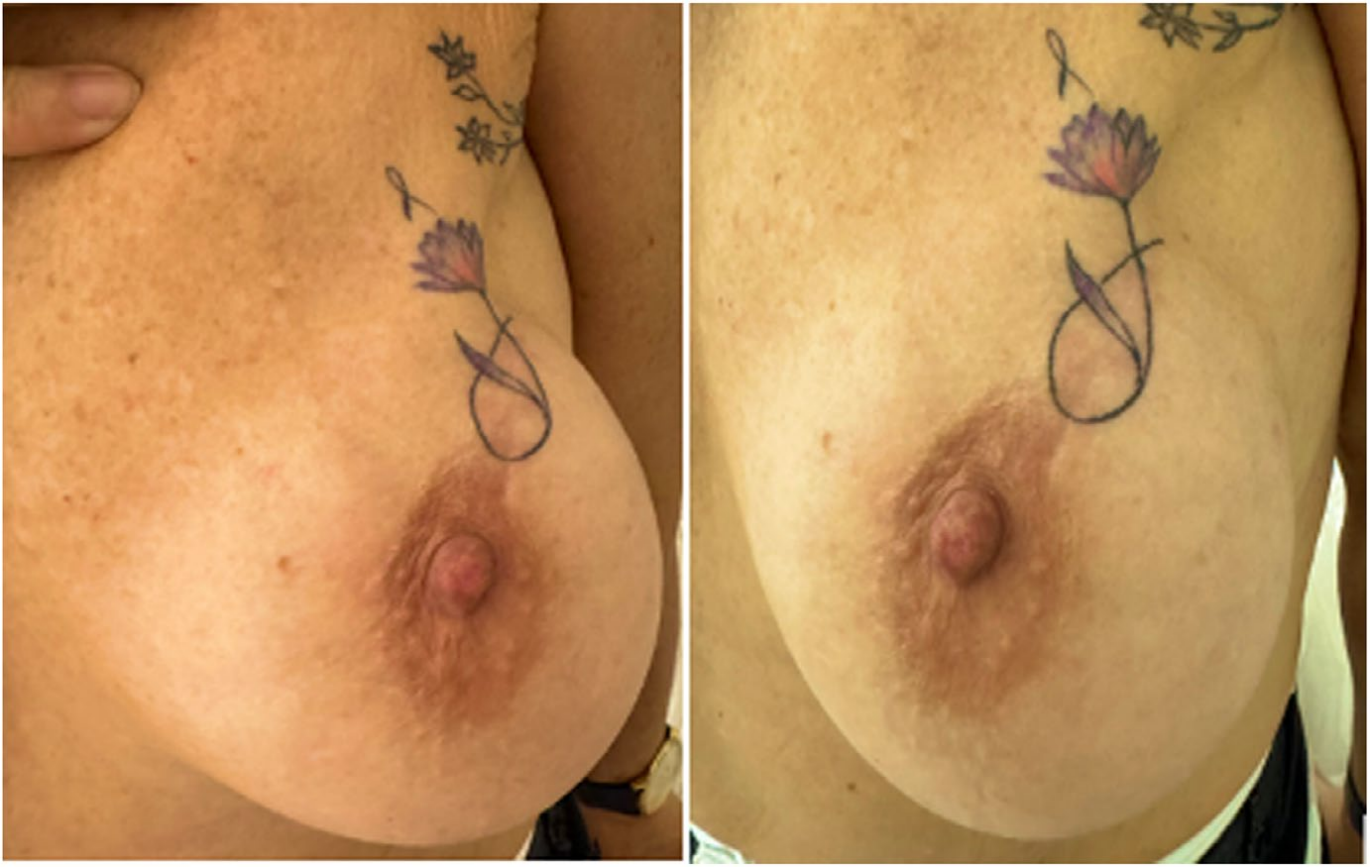
Before photo, left breast skin contracture and asymmetry after cancer surgery and radiotherapy are especially evident in the upper right quadrant of the areola.

The patient was initially evaluated in February 2024 (2 years after breast cancer surgery and radiation therapy), with complete recovery from the oncological condition. During the initial consultation, she reported feeling affected by the aesthetic appearance, which influenced her interaction with her husband. She felt as if she had cancer each time she looked in the mirror. The patient expressed an interest in pursuing nonsurgical treatment options. Physical examination confirmed visible breast asymmetry, with upper pole fullness and retraction of the skin on the left breast, especially on the areola, which appeared retracted. This asymmetry negatively affects patients' self‐esteem and quality of life (Figure 2). The patient was counseled about the risks and benefits of intradermal Botulinum Toxin A injections and requested to sign a written consent form to use their photographs and information for future medical publications.

The patient received intradermal injections of 100 IU of IncoBonTA (Xeomin Merz Pharmaceuticals GmbH, Frankfurt, Germany). The application followed a specific protocol according to skin contraction.

### 3.1 Treatment Protocol

**FIGURE 3:**
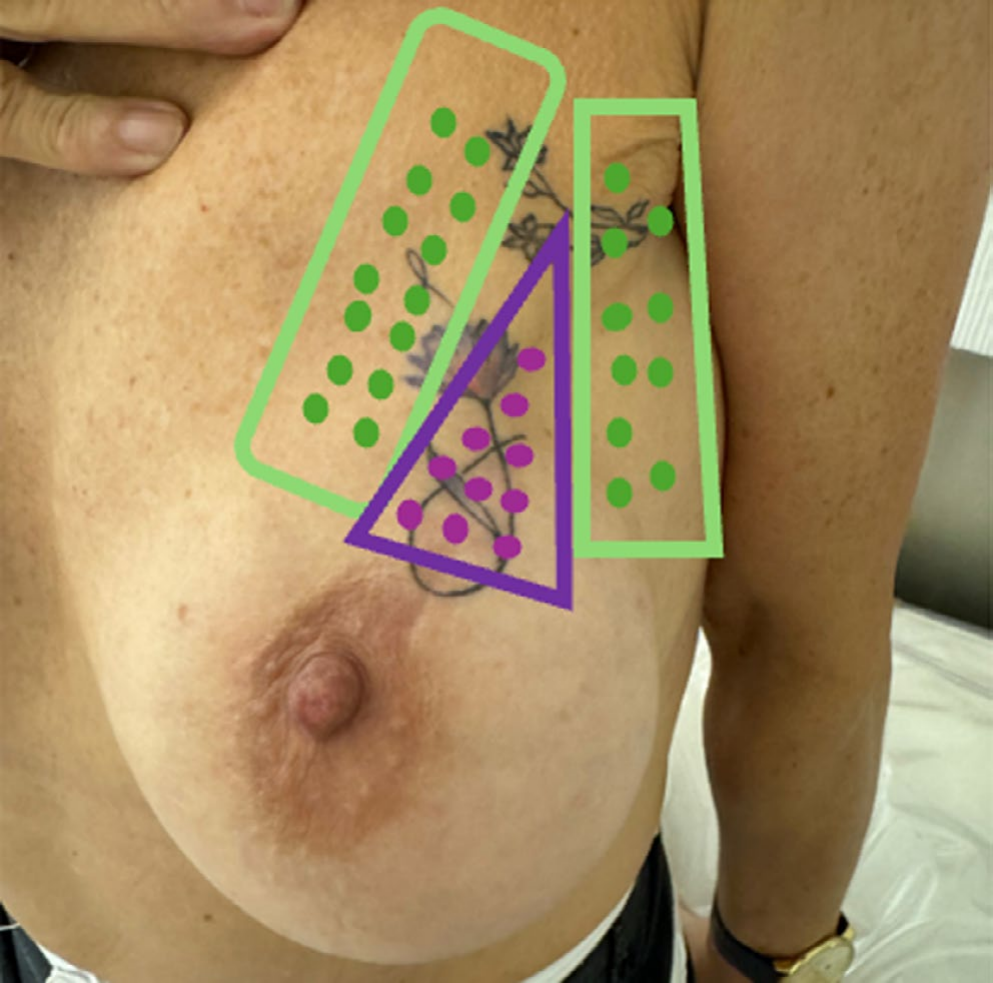
IncoBonTA areas of application: Two different dilutions were applied.

The patient underwent a series of intradermal injections of Inco‐Botulinum Toxin A across the affected area. The injection pattern follows the regions outlined in Figure 3.

A higher concentration solution with 50 units of IncoBonTA, hyperdiluted in 5 mL of standard saline solution (0.9%), was applied to the outer region (area of the green rectangles, external peripheral area), leaving approximately 0.02 IU of IncoBonTA intradermally per point, with approximately 0.5 cm between each point. In the central area (area of the purple triangle), close to the nipple and areola, a different dilution of 50 IU IncoBonTA in 2 mL of saline was used intradermally, with approximately 0.25 cm between each point.

The patient exhibited significant improvement in breast appearance at the 2‐week follow‐up visit. The upper pole fullness decreased, and skin retraction, particularly in the areola and nipple regions, was less pronounced.

**FIGURE 4:**
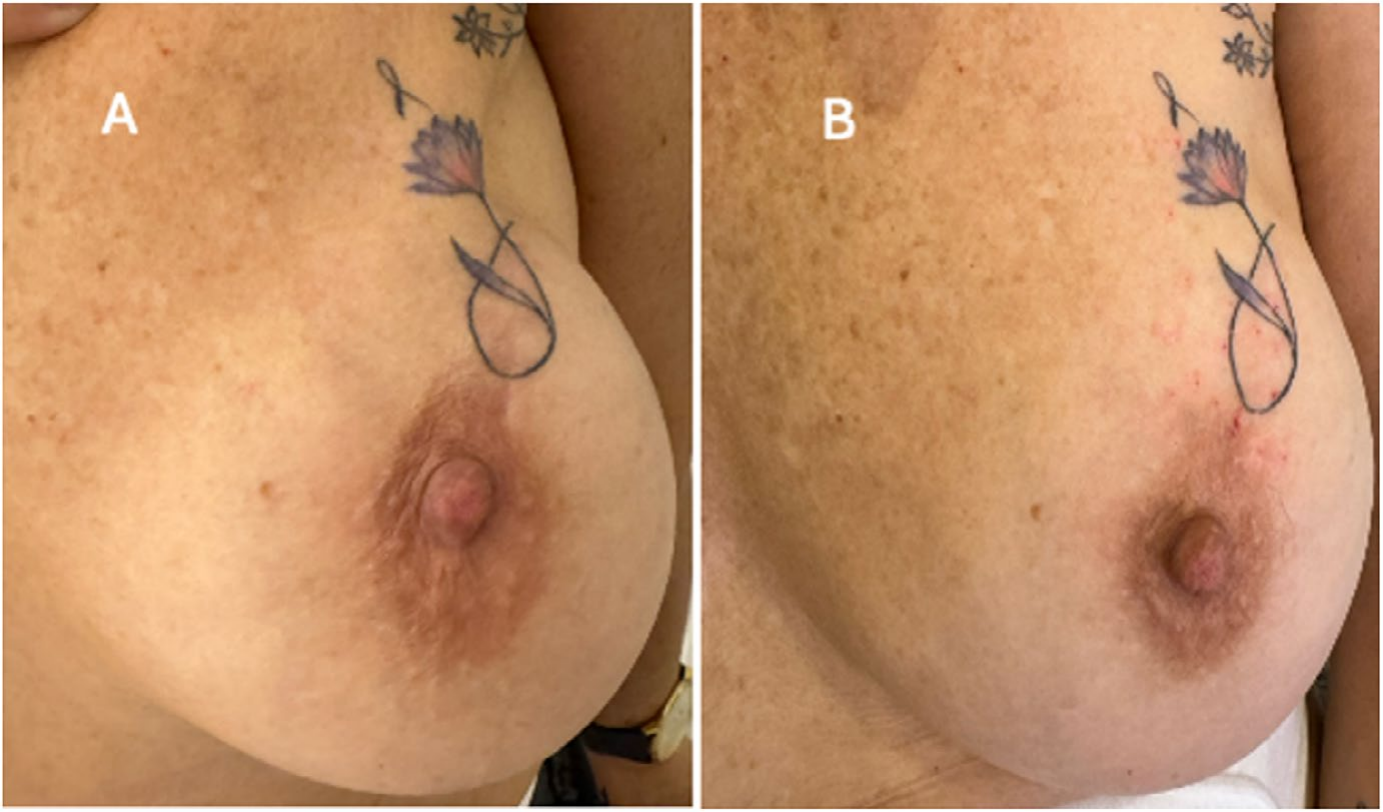
Improved symmetry and overall appearance 15 days post‐treatment (A: before, B: after). Additional periareolar toxin (20 IU) administered during the initial session (red dots visible).

The final follow‐up, 15 days postprocedure, demonstrated substantial improvement in upper breast asymmetry, notably in the areola and nipple areas (Figure 4). During this follow‐up visit, an additional 20 IU of IncoBonTA (Xeomin, Merz Pharmaceuticals GmbH, Frankfurt, Germany) was administered intradermally over the area of skin contracture as a touch‐up procedure.

The patient was delighted by the results, reporting a positive impact on her self‐esteem and quality of life. In conclusion, this case report demonstrates the potential of intradermal botulinum toxin injections as a minimally invasive and effective treatment option for addressing postsurgical breast asymmetry in breast cancer patients.

## 4 Discussion

Breast cancer is one of the most prevalent cancers affecting women worldwide [3]. Treatment typically involves surgery, radiotherapy, and chemotherapy, which have become increasingly influential in controlling cancer cell proliferation and, in many cases, curing the disease. However, these treatments can result in complications such as breast asymmetry, deformities, upper limb movement alterations, postsurgical pain, and contractures [4]. These adverse effects can significantly impact a patient's quality of life, self‐image, and mental and emotional health, making it essential for physicians to address these mental health consequences as part of cancer treatment [16].

The mechanism by which botulinum toxin manages postmastectomy sequelae is complex. By inhibiting the release of acetylcholine at neuromuscular junctions, botulinum toxin relaxes muscles, reduces contractures, and minimizes distortion of breast tissue. This relaxation can improve the aesthetic appearance of the breast, particularly in cases of asymmetry or retraction due to scarring or muscle tightness [11, 13]. By altering muscle contractions, IncoBonTA can enhance aesthetic outcomes.

Furthermore, botulinum toxin exhibits anti‐inflammatory properties that may help prevent capsular contracture, a common complication in breast reconstruction [9]. Capsular contracture, characterized by the hardening of the capsule that compresses the implant, leads to asymmetry. The application of BoNT‐A can alleviate muscle contracture and aid in the repositioning phase. Moreover, BoNT‐A injections are effective in relieving postmastectomy pain syndrome, particularly muscle spasticity and neuropathic pain, which patients often do not recall.

Our literature review of this case report reveals the significant evolution of botulinum toxin. Initially used for strabismus [17], it was serendipitously found to improve expression lines, leading to widespread use in dermatology and cosmetics [7]. Its applications have since expanded to include the management of muscular dystonia, spasticity [18], neurological disorders [19], hyperhidrosis [20], migraine [21], urological conditions [22], and trigeminal neuralgia [23], among others, benefiting many patients.

While it seemed that botulinum toxin had reached its potential as a treatment for expression lines, recent studies indicate its promising impact on cell proliferation [24, 25], with favorable in vitro results and effects on breast cancer [26].

## 5 Conclusion

The literature review indicates that botulinum toxin could enhance the appearance and well‐being of breast cancer survivors by improving their satisfaction and mental health after cancer treatment. However, current research reports do not definitively support its application in nipple and areola placement. Despite this, the authors see potential in this area, although there is a lack of data to compare the effectiveness of injections into the skin versus muscles and to determine the optimal dilutions and dosages for these specific body regions.

While botulinum toxin is not officially approved for this purpose, it is essential to consider investigating therapies for individuals who have survived breast cancer, as the emotional toll of this illness is often significant and closely linked to the presence of scars and physical abnormalities.

This report proposes that botulinum toxins could improve breast appearance by enhancing positioning, based on a study combining solutions with procedures to relax cutaneous layers. The authors involved in this study contributed valuable insights from their various areas of expertise, and a collaboratively conducted literature review informed the decision to perform the treatment using Incobotulinum (Xeomin Merz Pharmaceuticals GmbH, Frankfurt, Germany).

Furthermore, the application plane utilized in this case differs from those employed in other reports or scientific articles, where the toxin is typically administered at the pectoral muscle. However, further research is essential to establish effective injection techniques, dosages, and long‐term outcomes to fully leverage the use of botulinum toxins in addressing aesthetic concerns in breast cancer patients.

In conclusion, it is vital to expand the exploration of the different areas that can be treated with botulinum toxin and to continue uncovering its potential benefits.

## Author Contributions

All authors confirm their contributions to the paper as follows: Alejandra Bugallo, Andrea Marcela Parra, Luis Alberto Parra, Andreina Martinez Amado: study conception and design. Alejandra Bugallo, Victoria De la Fuente, Evalicia Murúa, Eliana Garcés: data collection. All authors: interpretation of results. Andrea Marcela Parra, Luis Alberto Parra, Andreina Martinez Amado: draft manuscript preparation.

## Ethics Statement

The authors confirm that the journal's ethical policies, as noted on the journal's author guidelines page, have been adhered to.

## Conflicts of Interest

The authors declare no conflicts of interest.

## Data Availability

The data that support the findings of this study are available from the corresponding author upon reasonable request.
